# Host-dependence of *in vitro* reassortment dynamics among the Sathuperi and Shamonda Simbuviruses

**DOI:** 10.1080/22221751.2019.1586410

**Published:** 2019-03-21

**Authors:** Damien Coupeau, Calixte Bayrou, Pierre Baillieux, Axel Marichal, Anne-Cécile Lenaerts, Céline Caty, Laetitia Wiggers, Nathalie Kirschvink, Daniel Desmecht, Benoît Muylkens

**Affiliations:** a Veterinary Department, Faculty of Sciences, Namur Research Institute for Life Sciences (NARILIS), University of Namur (UNamur)Namur, Belgium; b Department of Morphology and Pathology, FARAH Research Center, Faculty of Veterinary Medicine, University of LiègeLiège, Belgium

**Keywords:** Orthobunyavirus, reassortment, recombination, viral selection, arbovirus

## Abstract

Orthobunyaviruses are arboviruses (Arthropod Borne Virus) and possess multipartite genomes made up of three negative RNAs corresponding to the small (S), medium (M) and large (L) segments. Reassortment and recombination are evolutionary driving forces of such segmented viruses and lead to the emergence of new strains and species. Retrospective studies based on phylogenetical analysis are able to evaluate these mechanisms at the end of the selection process but fail to address the dynamics of emergence. This issue was addressed using two Orthobunyaviruses infecting ruminants and belonging to the Simbu serogroup: the Sathuperi virus (SATV) and the Shamonda virus (SHAV). Both viruses were associated with abortion, stillbirth and congenital malformations occurring after transplacental transmission and were suspected to spread together in different ruminant and insect populations. This study showed that different viruses related to SHAV and SATV are spreading simultaneously in ruminants and equids of the Sub-Saharan region. Their reassortment and recombination potential was evaluated in mammalian and in insect contexts. A method was set up to determine the genomic background of any clonal progeny viruses isolated after *in vitro* coinfections assays. All the reassortment combinations were generated in both contexts while no recombinant virus was isolated. Progeny virus populations revealed a high level of reassortment in mammalian cells and a much lower level in insect cells. *In vitro* selection pressure that mimicked the host switching (insect-mammal) revealed that the best adapted reassortant virus was connected with an advantageous replicative fitness and with the presence of a specific segment.

## Introduction

Several mechanisms generate diversity in multipartite viruses. Novel viral genotypes can be generated through mutations, recombination and reassortment. While mutations lead to a progressive evolution of viruses, the recombination and reassortment processes represent a form of genetic exchange that has the potential to provide many of the benefits of sexual exchange and accelerates the rate of acquisition of genetic traits that overcome adaptive host barriers [1,2]. Virus reassortment plays a key role in the emergence of new viruses. This process, exclusive to segmented viruses, leads to the generation of progeny viruses with novel genome combinations. These combinations derive from the shuffling of gene segments after co-infection of a host cell with multiple viruses. In contrast to reassortment, the recombination process occurs through an intragenomic template switch mechanism [3]. This can lead, as in the case of reassortment, to fundamental shifts allowing the emergence of a new virus. Both processes have been associated with changes in the host/vector tropism, escapes of the adaptive immune response and changes in pathogenicity [1,4–8]. In multipartite RNA viruses, reassortment and recombination rates are dependent on the virus family and genus. Within the *Bunyavirales* order a high reassortment level has been described [9]. In comparison, the recombination process is a rare phenomenon with only a few reports of recombination events described in natural Bunyavirus isolates [10–17].

The *Bunyavirales* order is a large order composed of nine families that infect mammals, insects or plants. The majority of these viruses are transmitted by different arthropod species but also by mammals in the *Hantaviridae* family. Since the replication process and encoded structural proteins are conserved, very few differences distinguish the different families. These enveloped viruses possess a genome composed of 3 segments of negative sense single-stranded RNA. The small (S) segment encodes the nucleoprotein N and the nonstructural protein NSs in an overlapping ORF. The medium (M) segment encodes the two glycoproteins Gn and Gc and the non-structural protein NSm. The large (L) segment encodes the RNA-dependent RNA polymerase (RdRp). The *Orthobunyavirus* genus belongs to the *Peribunyaviridae* family and includes numerous human or veterinary pathogens such as the viruses belonging to the Simbu serogroup. This group comprises at least 25 members that are currently sorted into seven species, namely *Manzanilla virus*, *Oropouche virus*, *Akabane virus*, *Simbu virus*, *Shuni virus*, *Shamonda virus* (SHAV) and *Sathuperi virus* (SATV).

The impact of reassortment in the evolution of *Bunyavirales* has been clearly demonstrated in many genera. It has been responsible for the emergence of several viruses. Briese et al consider the possibility that most if not all currently recognized Bunyaviruses represent reassortants, some of which are reassortants of existing viruses, and the others of extinct viruses [9]. In ruminants, several Simbuviruses have been associated with abortion, stillbirth and congenital malformation occurring after transplacental transmission. Two of these Simbuviruses SHAV and SATV have been reported to be present together in different contexts. After the initial report of SATV spreading in India in 1957, this Simbuvirus was detected in Nigeria between 1967 and 1970 from cattle blood [18] and from field populations of biting midges of the Culicoides genera [19]. The other species SHAV was initially isolated in Nigeria both from cattle [18] and from biting midges [19] during the same period. About 30 years later, the two viral species were detected in the Japanese archipelago. SATV was isolated from sentinel cattle in the Western part of the archipelago in 1999 [20] while SHAV was found in cattle and biting midges in the Southern part of Japan in 2002 [21]. Recent years have renewed interest in these two viral species. In 2011, a new virus emerged in Europe and rapidly spread in the ruminant livestocks causing severe economic losses. The aetiologic agent was named the Schmallenberg virus (SBV). Phylogenetic studies have indicated that SBV is a strain belonging to the SATV species [22]. Reassortment event(s) either shaped SBV after a coinfection involving SHAV and an SATV related virus [23] or shaped SHAV after a coinfection involving SBV and one unidentified virus [22]. The lack of viral isolates related to SHAV and/or SATV that emerged and spread over time from the 1960s to 2011 impaired establishing a precise scenario regarding SHAV, SATV and SBV evolution.

In order to provide with a prospective view of the gene shuffling that might occur when related Simbuviruses are spreading in an animal population, we decided to investigate reassortment and recombination capacities of Orthobunyaviruses through experimental co-infections involving the original SHAV and SATV isolates. After showing that different viral species related to SHAV and SATV are spreading simultaneously in animal populations of the Sub-Saharan region, we decided to address the plasticity of the two viral species through reassortment and recombination. *In vitro* co-infection experiments were performed in two relevant contexts of Bunyavirus infection: the mammalian and the insect contexts. This study reports on the dynamic rise and the selection of most adapted viral combinations obtained from gene mosaicism produced from two ruminant Simbuviruses.

## Results

### Epidemiological evidence of animal populations co-infected with two Simbuviruses related to SHAV and SATV in Sub-Saharan Africa

**Figure 1.**
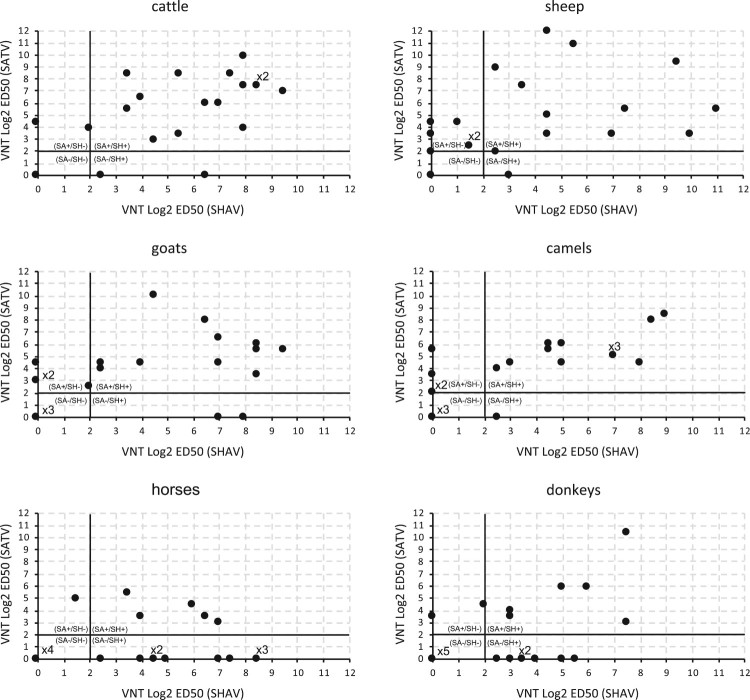
Viral Neutralization Test against SHAV and SATV performed in animal sera collected in northeastern Nigeria. Results are expressed in Log2 ED50 against SATV and against SHAV. Results are considered positive if Log2 ED50 > 2. Four ruminant species (Cattle, Goats, Sheep and Camels) and two equine species (Horses and Donkeys) were analysed. Results obtained for 20 individuals of each animal species are presented. When identical serologic results were observed for several animals, the number of repeats is indicated above the dot.

The two viral species SHAV and SATV used in this study have been suspected of circulating in Africa [24–27]. In this context, we performed a retrospective serological analysis to identify the presence of anti-Simbuvirus antibodies in blood samples collected in northern Nigeria in 2009 on 1525 animals from 6 species (4 ruminants and 2 equids). This analysis revealed the presence of a high rate of Simbuvirus circulation, since 18–59% of animals were seropositive in an ELISA assay using the SBV N protein as a capture antigen (ID Screen^®^ Schmallenberg virus Competition Multi-species, ID.vet). To better determine the serological status of these animal populations, a set of 20 randomly selected samples was tested using a Virus Neutralisation Test (VNT) in each animal species against SHAV and SATV. These VNTs showed that a very high proportion of animals were positive towards either SHAV, SATV and/or closely related viruses. Indeed, 87% (104/120) of sera contained antibodies neutralizing either SHAV or SATV (Figure 1).

Several sera showed high neutralization capacities towards SHAV while they failed to neutralize SATV and vice-versa (Figure 1). This confirmed that no cross-neutralization exists between the two viral species [18]. This suggests that animals showing neutralizing antibodies towards SHAV and SATV were infected either by two viral species, one related to SHAV and the other related to SATV, or by a single viral species that cross-reacts with SHAV and SATV. Considering the first scenario, 57% (69/120) harboured antibodies against both a SHAV related virus and an SATV related virus (Figure 1). The ratio of double positivity in the VNT was higher in ruminants (70%, 56/80) compared to equine species (32%, 13/40). The co-circulation of two Simbuviruses serologically related to SHAV and SATV in the same country and in several animal populations supports potential situations of co-infection with viral species related to SHAV and SATV. In order to analyse the consequences of such co-infection we selected SHAV and SATV as parental strains and investigated their genetic exchange capacities.

### Selection of two Simbuviruses as parental species for co-infection experiments

**Figure 2.**
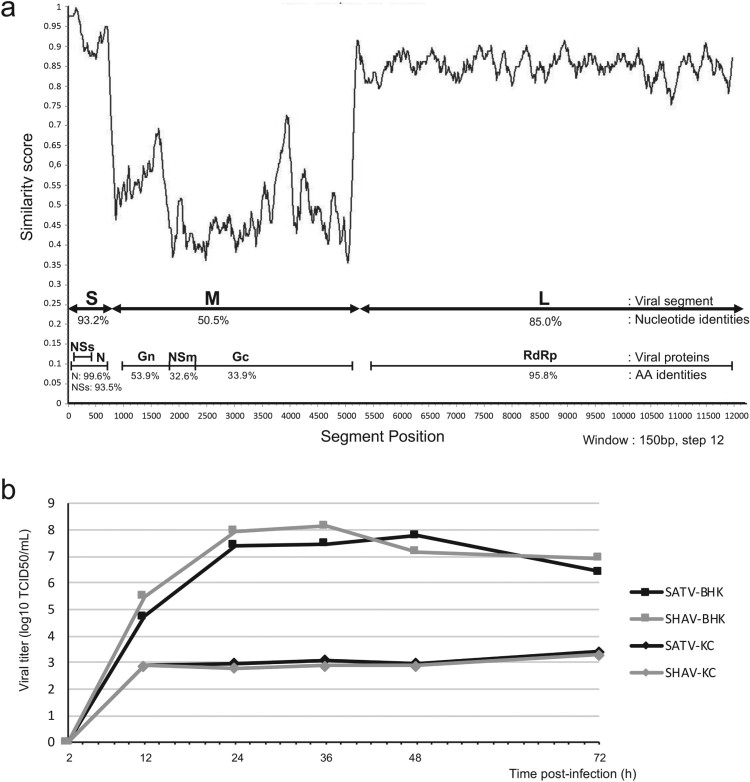
Comparison of the sequences and growth kinetics of the two Simbuviruses SHAV and SATV. (a) Similarity plot based on the aligned genome sequences of SHAV and SATV. The plot was performed using the Recombination Analysis Tool (RAT) [48]. Segments S, M and L are indicated by double arrows and encoded proteins are illustrated below. Percentage of nucleotide and protein identity for each segment and each encoded protein are annotated. (b) Growth kinetics of the two viruses SHAV and SATV in mammalian and in insect cells. Titers, expressed in TCID_50_/mL, were determined after 2, 12, 24, 36, 48 and 72 h post infection at MOI 0.01. SATV growth kinetics are illustrated by black lines and SHAV growth kinetics are indicated by grey lines. Growth curves were tested in BHK-21 cells (square lines) or in insect KC cells (diamond lines).

In order to assess sequences similarities among ruminant Simbuviruses, a similarity plot analysis was performed on the SHAV and SATV sequences (Figure 2(a)). Sequence alignment and comparison were carried out both at the nucleotide and protein levels and revealed that S and L segments were more conserved than the M segment (Figure 2(a)). Proteins encoded from S and L segments showed identity scores from 96 (RdRp) to 99% (N) whereas proteins encoded from the M segment showed identity scores from 33% (NSm) to 54% (Gn).

To assess the adequacy of SHAV and SATV for use as a parental species in co-infection assays, growth kinetics were performed in mammalian and insect cell lines. As illustrated in Figure 2(b), no significant difference was observed between the two Simbuviruses whatever the cellular context. A comparison of replication capacities between the two cellular contexts showed a dramatic difference, with a high replication capacity for both viruses in mammalian cells compared to insect cells. Indeed, titres appeared 5 log10 higher in mammalian cells compared to insect cells (Figure 2(b)).

### Point mutations discriminable by specific PCR assays are suitable as reassortment and recombination markers

**Figure 3.**
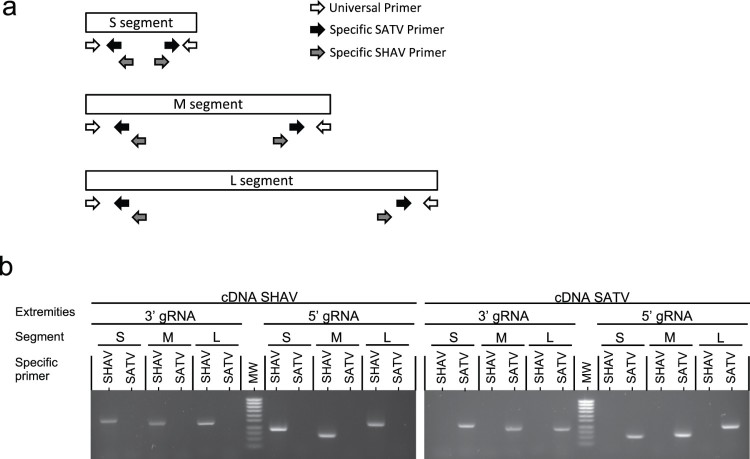
Method used to discriminate progeny viruses. (a) Schematic representation of primers used to discriminate both extremities of each segment. Universal primers are represented by white arrows, SATV specific primers are represented by black arrows and SHAV specific primers are represented by grey arrows. (b) Electrophoresis patterns of discriminative PCRs. Each segment (S, M and L) of both viruses (SHAV and SATV) were discriminated at both extremities (3′gRNA and 5′gRNA). On the left: SHAV and on the right: SATV. MW: Molecular Weight (Smart ladder, Eurogentec).

In order to differentiate the parental origin of any segment in the new progeny viral population obtained after SHAV and SATV co-infection, discriminant PCR assays for SHAV and SATV were set up. Primer design was based on Single Nucleotide Polymorphims (SNPs) found in S, M and L segments of the two parental viruses (Figure 3, Table. S1). This approach aimed to identify the parental origin at both extremities of each segment and thus determine the genetic background of any progeny viruses (Figure 3(a)).

After validating their accuracy and their specificity (Figure 3(b)), these sets of discriminative PCR assays were used to determine the genotype of any viruses isolated in this study. A progeny virus was sorted as parental (SHAV or SATV) if genotyping was fully concordant for the three segments (two possibilities) and as reassortant (R1 to R6) if genotyping of any segment was discordant with the other segments (2^3^−2 = 6 possibilities). To detect the occurrence of recombination, the extremities of M and L segments were used as recombination markers. Their genotype was determined as belonging to the SHAV or SATV genotype to detect any discordance that would result from odd number of recombination event(s).

### Generation and characterization of progeny viruses obtained after co-infection in mammalian and insect cells

In order to determine the exchange capacities of SATV and SHAV through reassortment or recombination, co-infection assays were performed in mammalian and in insect cells. Two concentrations of parental viruses were used to co-infect both cell types to analyse the impact of the viral load on the reassortment/recombination processes.

**Figure 4.**
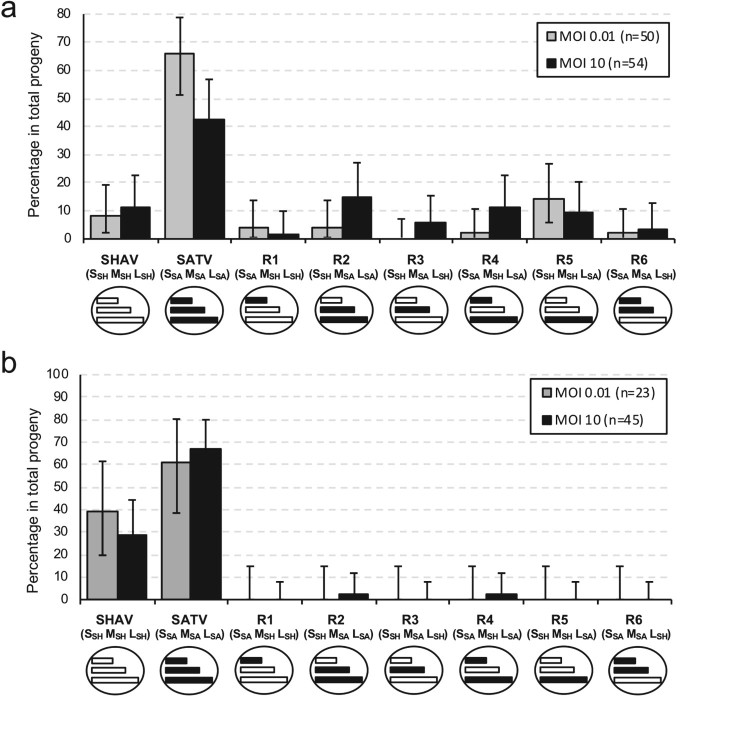
Distribution of progeny viruses obtained after co-infection experiment in BHK-21 mammalian (a) and insect (b) cells (passage 0). Gray boxes represent frequency of progeny viruses obtained after co-infection at MOI 0.01 and black boxes represent frequency of progeny viruses obtained after co-infection at MOI 10. Parental and reassortant viruses and theirs respective genotypes (S, M, L) are indicated below each box and are illustrated by schematic viruses with coloured segments (white for SHAV segment and black for SATV segment). Error bars represent the confidence intervals of each virus frequency (*α* = 0.05).

In mammalian cells co-infected at high (10) and low (0.01) multiplicity of infection (MOI), around 50 progeny viruses were isolated and genotyped under each condition (Figure 4(a)). Under high MOI, all the reassortment combinations were identified with a high rate of reassortment (43%) with 2–15% of each combination. Even under a low level of co-infection, five of the six reassortment combinations were identified with a global reassortment rate of 26%. Whatever the viral load, the two parental viruses were identified in the progeny with a higher rate for SATV than for SHAV. At high MOI, two reassortants were found at an equal or higher frequency than the parental virus SHAV, namely the reassortant R2 (S_SH_ M_SA_ L_SA_) and the reassortant R4 (S_SA_, M_SH_, L_SA_). At low MOI, one combination was more frequently isolated than SHAV, namely the reassortant R5 (S_SH_, M_SH_, L_SA_). These three reassortants possess the L segment of SATV.

To test the impact of the cellular context, co-infections were performed in KC cells (Figure 4(b)). In the insect cell line, reassortment frequency was drastically reduced when compared with the mammalian context. Under low MOI (0.01), no reassortant was isolated. Under high MOI (10), two reassortant combinations were found: the reassortant 2 (S_SH_ M_SA_ L_SA_) and the reassortant R4 (S_SA_, M_SH_, L_SA_). As observed in the mammalian cells, the two parental combinations were observed in the co-infection supernatant with a higher proportion of SATV.

### Phenotypic characterization of the different reassortants

In order to confirm the clonality and the stability of the different genetic combinations, one clone of each reassortant type was individually propagated during 10 successive passages in BHK-21 cells. After 10 passages, genomic pattern was checked by the whole set of PCR genotyping assays and was found conserved for each reassortant. In addition, all the reassortants were still able to induce cell lysis at the end of the procedure (data not shown). Thus all the reassortants obtained by co-infection with the two parental viruses SHAV and SATV were clonal and remained infectious.

**Figure 5.**
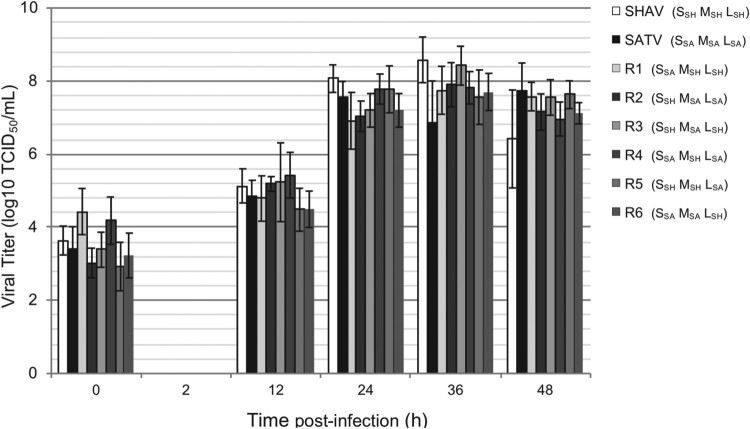
Growth kinetics of parental and reassortant viruses in BHK-21 cells. Titers, expressed in TCID_50_/mL, were determined after 0, 2, 12, 24, 36 and 48 h post infection at MOI 0.01 in BHK-21 cell line. Error bars represent standard deviation calculated by using triplicate experiments carried out for each parental or reassortant virus.

The phenotype of the reassortants and parental strains was firstly studied by comparing growth kinetics in mammalian cells (Figure 5). The different reassortants and the parental viruses displayed similar replication curves with no significant difference observed at any time point of the infection through an ANOVA2 analysis.

**Figure 6.**
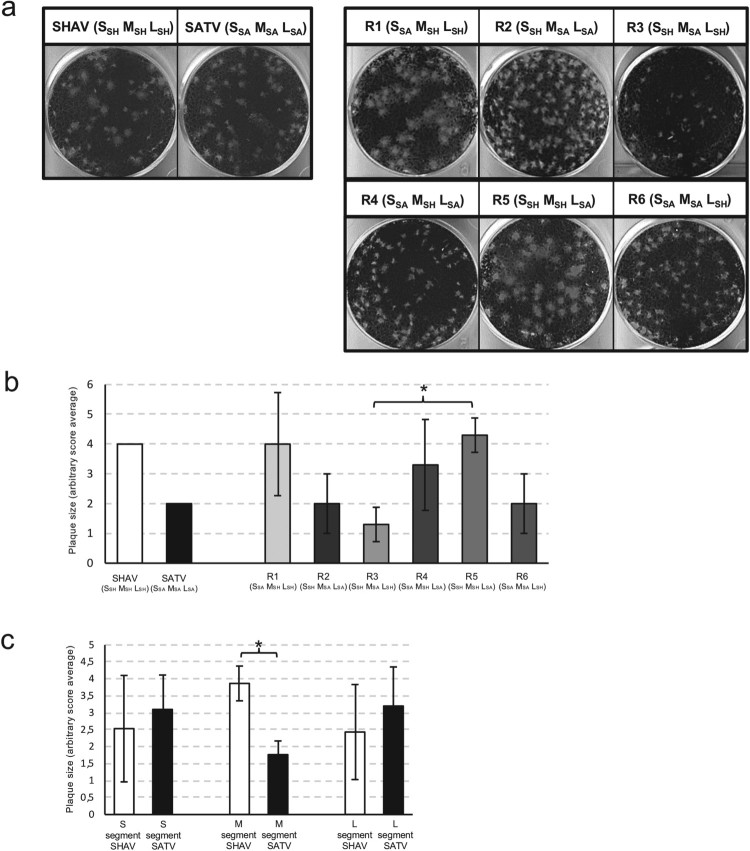
Plaque size assays of parental and reassortant viruses. (a) Examples of plaques obtained after infection of BHK-21 cells with dilutions of SHAV, SATV and the six different combinations of reassortants (R1 to R6). (b) Schematic representation of plaque size induced by the parental or the reassortant viruses. Plaque size value of the parental strains SHAV and SATV were fixed at 4 and 2 respectively. Error bars represent standard deviation calculated by using three different sets of viruses. An ANOVA test was performed to compare the six reassortants together. A Tukey test was performed to compare each reassortant against every other reassortant. * represents significant difference with *P* < .05. (c) Comparison of the average plaque sizes induced by the different reassortant viruses according to the parental origin of each segment. Error bars represent standard deviation calculated by using three different viruses harbouring this segment. * represents significant difference with *P* < .05 by using Welch’s *t*-test.

Phenotype characterization was completed by plaque size assays. Three independent sets of the six different reassortant combinations were tested. In a first assay, the repeatability of the plaque size assays was checked by analysing a first set of six reassortants (Set 1 in Figure S1) in independent triplicate. Since similar data were observed, the assay repeatability was demonstrated (data not shown). The other two reassortant sets (2 and 3) were tested once. Plaque sizes induced by any reassortant were compared with the plaque sizes of the two parental viruses by using the Kolmogorov–Smirnov statistical test (Figure S1) and scores were given according to the plaque size associated with each reassortant and parental virus. As illustrated in Figure 6, the plaque size analysis revealed significant differences. Scores comparison showed that R5 (S_SH_, M_SH_, L_SA_) produced the largest plaques and R3 (S_SH_, M_SA_, L_SH_) the smallest (*p* = .039) (Figure 6(b)). In order to determine whether segment origin is involved in this phenotype, the plaque size scores were analysed according to the parental segment origin (Figure 6(c)). When considering the S and L segments, no difference was observed in the plaque size data, but the comparison showed a significant difference when the M segment was considered. Indeed, viruses possessing the M segment from SHAV (R1, R4 and R5) produced larger plaques in comparison with those possessing the M segment from SATV (R2, R3 and R6). Strikingly, the mean score of viruses harbouring the SHAV M segment is almost 4 and the mean score of viruses harbouring the SATV M segment is around 2. These values corroborated scores given to the parental strains indicating that plaque size phenotype is dependent on the origin of the M segment.

### Competition assays among viral mixtures propagated after initial co-infection of mammalian and insect cells

As demonstrated above, co-infection involving two related Simbuviruses generated a high level of diversity in the viral population after an extensive shuffling of viral segments. Yet, this massive exchange did occur in BHK-21 cells but not in insect cell lines. Since different phenotypes were associated with parental and reassortant viruses when cultivated separately, we decided to investigate viral competitive and replicative fitness from the viral mixtures that were obtained after initial co-infections in mammalian and insect cells. We wanted to determine if more favourable segment combinations might drive the selection of more adapted reassortants under the tested conditions. In order to mimic the host switching observed in the natural cycle of an arbovirus infection, progeny viral populations were used to successively infect cells by alternating the insect and the mammalian context.

**Figure 7.**
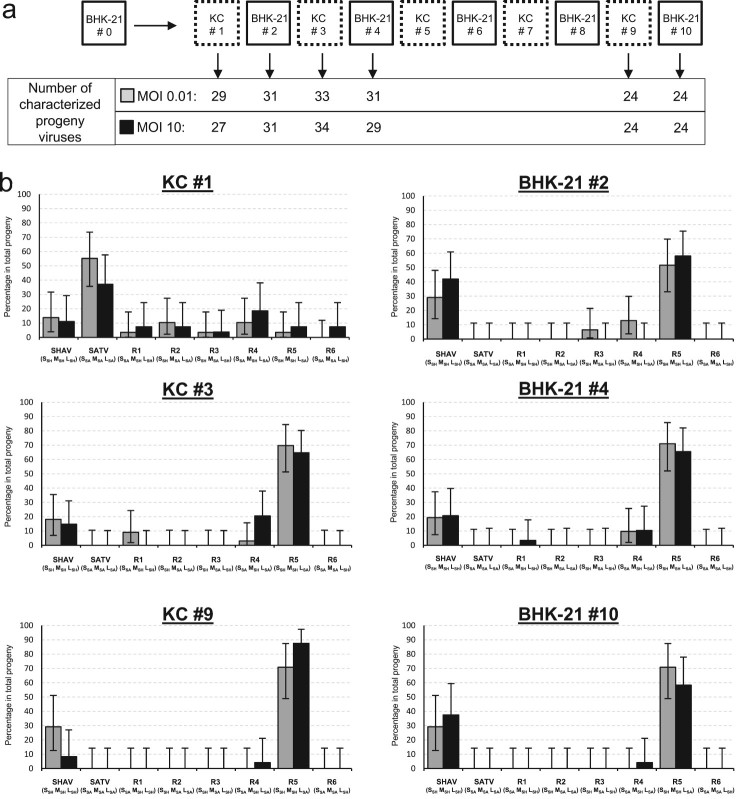
Dynamics of reassortment observed during 10 successive infections by alternating the insect and the mammalian context. (a) Schematic representation of the competition assay where the initial viral population was alternatively propagated in insect and mammalian cells during 10 passages. The number of characterized progeny viruses for each tested MOI are indicated for each analysed passage (#1, #2, #3, #4, #9 and #10). (b) Representation of the viral population identified for the two tested MOI: 0.01 (grey boxes) and 10 (black boxes). Parental and reassortant viruses and their respective genotypes (S, M, L) are indicated below each box. Error bars represent the confidence intervals of each virus frequency (*α* = 0.05).

Initial infectious supernatants (#0 = passage 0) obtained from co-infection assays in mammalian cells at MOI 0.01 and 10 were alternatively propagated in insect and mammalian cells during 10 passages (Figure 7(a)). Progeny viruses were isolated and analysed at several passages (#1, #2, #3, #4, #9 and #10); passages 1, 3 and 9 corresponding to progeny virions obtained after insect cells infection and passages 2, 4 and 10 to progeny virions obtained after mammalian cells infection. Whatever the tested MOI, the viral population obtained after the first passage in insect cells (Figure 7(b)) displayed a similar pattern to the one observed after initial coinfection in the mammalian cells (Figure 4(a)). A high diversity was still present in the viral population and only the reassortant R6 was lacking in the situation produced from the low MOI. After the second passage occurring in mammalian cells, a stringent selection was observed (Figure 7(b)). Three viral combinations (SATV, R2 and R6) disappeared and were no more detected. At the third passage, the reassortant R3 that harbours the SATV M segment disappeared. Altogether, all the viruses (SATV, R2, R3 and R6) containing the M segment of SATV vanished quickly from the viral population (Figure 7(b)). In an opposite way, two viruses were selected in the viral population from #2 to #10, the parental virus SHAV and the reassortant R5 (S_SH_ M_SH_ L_SA_). Regarding the last two combinations (R1 and R4), the R1 was found until the passage 4 and the R4 was detected at low level throughout the competition assay (Figure 7(b)). Altogether, this approach mimicking the host switching to propagate *in vitro* a viral population for several generations cleared the viral combinations possessing the M segment inherited from SATV. The virions possessing the M segment of SHAV were best adapted especially the reassortant R5.

**Figure 8.**
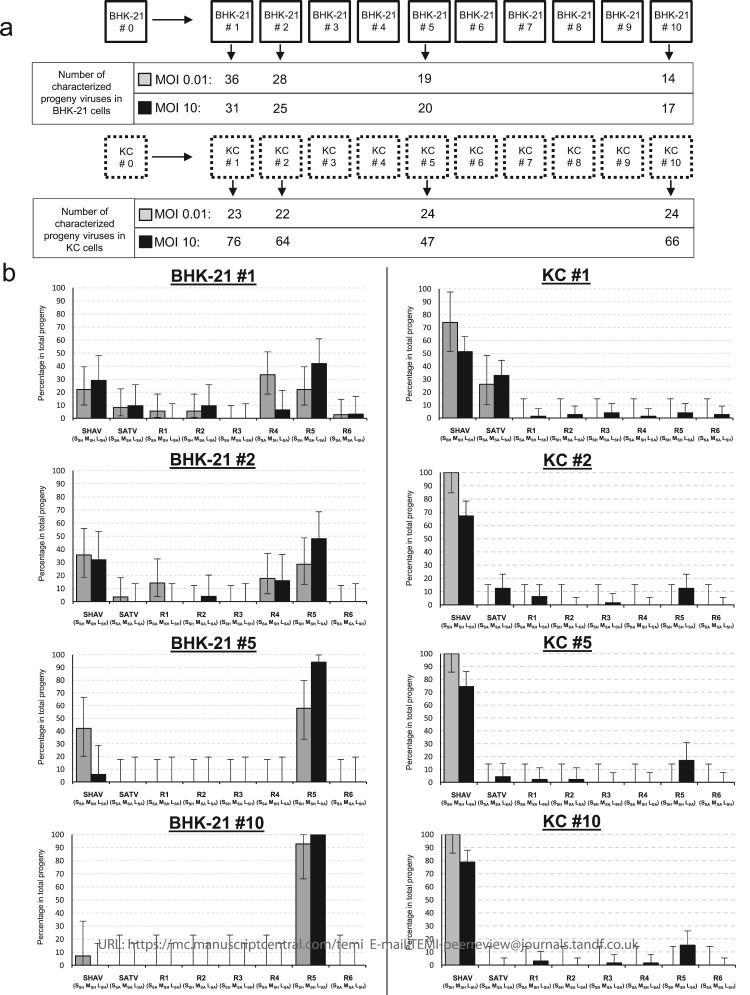
Dynamics of reassortment observed during 10 successive infections in mammalian or in insect cells. (a) Schematic representation of the competition assay where the initial viral populations were propagated in insect or in mammalian cells during 10 passages. The number of characterized progeny viruses for each tested MOI are indicated for each analysed passage (#1, #2, #5 and #10). (b) Representation of the viral population identified in the two BHK-21 (left panel) and KC (right panel) cell lines and for the two tested MOI: 0.01 (grey boxes) and 10 (black boxes). Parental and reassortant viruses and their respective genotypes (S, M, L) are indicated below each box. Error bars represent the confidence intervals of each virus frequency (*α* = 0.05).

In order to explore the impact of each cellular context on the selection that occurred on the viral population submitted to alternative passages, competition assays were carried independently in both cellular contexts (Figure 8). Competition assays in mammalian cells ended up selecting the reassortant R5 (S_SH_ M_SH_ L_SA_). The latter overtook the seven other genetic combinations including the two parental viruses (Figure 8(b)). Competition assays carried out in the insect cells (Figure 8(b)) showed a very different evolution pattern when compared with the mammalian context. Although reassortment was a very rare event in the initial co-infection in insect cells co-infected with an MOI of 10 (Figure 4(b)), reassortment mosaicism did arise over time and some combinations were maintained when infection was perpetuated for 10 passages (Figure 8(b)). Even a higher proportion of the reassortant R5 over to others reassortants was observed, these results revealed a weaker selection in the progeny population in insect cells that actually favours the persistence of a more diversified viral population over time. In the progeny, viral population propagated independently in the insect cell line, one of the parental species SHAV was selected over the other one.

The comparison of the different conditions tested for competition assays revealed concordance between assays. Indeed, alternative passages in insect and mammalian contexts led to the selection of two combinations (R5 and SHAV) while R5 was best-adapted progeny virus selected in mammalian cells and SHAV best-adapted virus selected in the insect cells.

### Recombination events

In parallel to reassortment analysis, the occurrence of intra-molecular recombination was assessed. In order to determine the occurrence of this phenomenon, both extremities of M and L segments were analysed on the viral clones isolated from successive infections of mammalian and insect cells. We decided to focus on situations where high levels of variability were detected. Thus all virions isolated at passages 0, 1 and 2 in BHK-21 cells, and at passages 1 and 2 in KC cells were characterized for intra-molecular recombination. Altogether, the recombination occurrence was sought for 556 progeny viruses. Both extremities for all the tested progeny viruses indicated the same parental origin for each characterized segment. This result showed that no intra-molecular recombination occurred under these conditions. Although the presence of recombinant viruses cannot be excluded under test conditions, the superior limit of the confidence interval indicates that the frequency of the phenomenon is lower than 0.66%.

## Discussion

Reassortment and recombination play a major role in viral evolution and in the rise of new viral species. Retrospective studies based on phylogenetical analysis are able to evaluate these mechanisms at the end of the selection process but fail to address the dynamics of emergence. We addressed this issue by using two Simbuviruses that are intimately connected with the history of a Bunyavirus that recently colonized European ruminant livestock, the SBV. Our approach was to carry out *in vitro* coinfection assays in both the mammalian and insect contexts to determine the reassortment and recombination capacities of these viruses. We found that several Simbuviruses circulate in ruminants and equids in the Sub-Saharan region. In a first scenario explaining the serological status of animal populations, three viral species are spreading, a SHAV related virus, an SATV related virus and a virus that cross-reacts with SHAV and SATV (unidentified yet). In a second scenario, animals showing neutralizing antibodies towards SHAV and SATV were infected by two Simbuviruses, one related to SHAV and one related to SATV. These animal populations supporting both viral infections provide adequate niches for the emergence of new viral combinations by reassortment and recombination. Altogether, *in vitro* coinfection with the two ruminant Simbuviruses SHAV and SATV generated a high diversification through reassortment while no recombination was observed. *In vitro* selection pressure that mimicked the host switching (insect-mammal) revealed that the best adapted reassortant could be connected with an advantageous replicative fitness.

Reassortment capacities in the *Bunyavirales* order was mainly assessed by the analysis of field strains isolated in naturally infected populations [28–34]. Only a few data are available from controlled co-infections carried out *in vitro* [35,36] or *in vivo* [37,38]. Reassortment analyses of natural Bunyavirus isolates or of progeny viruses obtained from *in vitro* experiments have revealed a non-random distribution of segment reassortment. Indeed, the majority of reported reassortants in the *Bunyavirales* order arises after the exchange of the M segment from one parental virus and the S and L segments from the second parental virus [9]. This partitioning pattern during reassortment in the circulating Bunyaviruses might be the consequence of different factors implicated in the reassortant selection. As observed for other segmented viruses, the segment assortment in the progeny viruses is probably dependent on RNA-RNA, protein–protein and RNA-protein interactions [2]. Moreover, the selection of a new combination acts at different levels: during progeny virion formation and genomic RNA packaging, during the extracellular life of the virus, and during infection/replication in a new cell or a new host [2].

In addition to these factors, the impact of the cellular context raises an important question for arboviruses. The preferred location for the reassortment of Bunyaviruses is still unknown. It has been postulated that it occurs preferably in the arthropod because replication in mammals is relatively brief, while arthropods remain infected throughout their lifetime. However, which kind of arthropod also seems important because the lifespan length, the number and the frequency of blood feeding differs.

In this study, although a higher reassortment frequency has been observed in mammalian context, all reassortment combinations have been identified in both contexts. Competition assays revealed a stronger selection in mammals compared to that in insects. Indeed, after 10 passages, only one type of virus has been identified in mammalian cells and five combinations have been found in insect cells.

Non-lytic infection in insect cells can foster dissemination; however, due to the interference mechanism that also exists in insects [39], blood feeding on two different animals infected with two different viruses needs to be close in time. If the two viruses do not infect the same vector (Culicoides versus mosquito), reassortment may occur exclusively in the mammal host and may induce a tropism shift of the putative reassortant. For viruses of the *Bunyavirales* order, the reassortment process is likely to occur in both host and vector.

Assays performed in this study were carried out with different environmental restrictions that have an impact on reassortment selection. The only criterion that had an effect was the origin of the M segment. Indeed, the M segment inherited from SHAV induced the “large plaque” phenotype and favoured the emergence of associated reassortants especially in mammals. In the opposite way, the origin of the S or L segment had no impact. In addition to its role in the selection of reassortants, other types of phenotypic shift induced by the transfer of the M segment through reassortment have been described in two families of the *Bunyavirales* order, the *Nairoviridae* and the *Tospoviridae*, where pathogenicity or vector transmission respectively were increased [40,41], although no effect was observed in two studies carried out in the *Hantaviridae* family [35,42].

Although the conditions are far from the field ecological niche of Simbuvirus evolution, the present study reports the high diversification occurring after coinfection situations involving related Simbuviruses. In addition, the successive infections propagated in the different contexts (mammal vs insect) offer a model for the selection of the most adapted virus in mammals, with the SHAV M segment conferring the best fitness under the tested conditions. However, different selective pressures were not taken into consideration in our assays carried out *in vitro*. The immune pressure directed against one of the parental viruses can modify the selection of reassortants, and notably the selection of the M segment. Since the latter encodes viral glycoproteins, neutralizing antibodies mainly raised against these envelope proteins may drive the selection of specific reassortant possessing a new set of these proteins. In a second aspect, compared to *in vitro* experiments, *in vivo* reassortment capacities seem to be limited by spatial and cellular diversity within the host due to the fact that viruses are not uniformly distributed in a host [43]. Another factor impacting the *in vivo* selection of arboviruses is related to the need of any progeny virion to remain infectious for both the host and the vector.

Others parameters can influence the proportion of reassortant in the progeny. The main parameter is the MOI. In influenza viruses, for example, Marshall et al. show an increase in reassortant proportion during *in vitro* experiments by using higher MOI [44]. In our assays concerning mammals, the MOI also increased the proportion of reassortants without affecting the spectrum of combinations that arose (Figure 8 & Figure S2). Because of the lower replication rate observed in insects, the MOI had even a more significant impact and no reassortant was detected at low MOI.

In this study, no recombinant virus was detected among 556 progeny viruses. Although we cannot say that the phenomenon does not exist (only single or odd number of recombination events can be detected using this method), its occurrence in Simbuviruses is very unlikely. There are currently two major hypotheses regarding the contribution of recombination to the evolution of multipartite RNA viruses. In the first, it is considered a minor mechanism for the evolution of multipartite RNA viruses and in the second, it is considered a major mechanism for the evolution of multipartite RNA viruses. In these viruses, the reassortment and recombination rate is dependent on the RNA polarity, on the virus family and genus studied, but it can also differ from strain to strain, within general a higher level of reassortment in comparison with recombination. In the case of Bunyaviruses, a high rate of natural genetic reassortment has been observed and few recombination events have been predicted by bioinformatics analyses but never obtained after in vitro coinfection. Some examples of predicted recombination in the *Bunyavirales* order exist [10–17]. However, due to its low rate of occurrence in comparison with that of the reassortment process, it seems not to be a major parameter for the evolution of this virus family.

In conclusion, the reassortment of RNA segments (genetic shift) complements genetic drift (accumulation of point mutation) as a powerful mechanism underlying the evolution of the Bunyaviruses and compensates for the low level of recombination in these viruses. However, reassortment is a multifactorial phenomenon which depends on the host, the vector, the two implicated viruses and the environment, with non-universal viral selective criteria that can vary from one case to another.

## Materials and methods

### Cells and viruses

BHK-21 fibroblasts (ATCC CCL-10) were maintained in Glasgow Modified Eagle’s Medium (GMEM) supplemented with 10% Foetal Bovine Serum (FBS), tryptose phosphate broth (2.95 g/L), Penicillin (100 U/mL) and Streptomycin (100 µg/mL). BHK-21 cells were cultured at 37°C in an atmosphere containing 5% CO_2_.

KC cells, obtained from *C. sonorensis* larvae, were maintained in Schneider medium (Gibco) supplemented with 10% FBS, amphotericin B (2.5 µg/ml) and gentamycin (25 µg/ml). KC cells were cultured at 25°C without additional CO_2_.

Two Orthobunyaviruses from the Simbu serogroup were used in this study: the Shamonda virus (SHAV) isolate Ib-An-5550, and the Sathuperi virus (SATV) isolate I-11155. Both viruses were adapted in BHK-21 cells and viral stocks were prepared from these cells. In order to obtain a concentrated inoculum, ultra-centrifugations were performed. After 36 h of infection, the supernatant was harvested and firstly centrifuged at 1000 g to remove cellular fragments. Then, the supernatant was ultra-centrifuged at 100,000 g by using a 30% sucrose cushion. The viral pellet was re-suspended in PBS, aliquoted and stored at −80°C.

### Blood sample collection and detection of Simbuvirus antibodies

In order to determine the presence of anti-Simbuvirus antibodies in African blood samples, sera from 1525 animals of 6 species (4 ruminants: cattle, sheep, goat, camel and 2 equids: horse and donkey) were collected in North Nigeria in 2009. Blood samples were obtained at slaughterhouse by the virus research laboratory (Prof S S Baba, University of Maiduguri, Nigeria).

Firstly, the blood samples were centrifuged at 1100 g for 25 min at room temperature to obtain serum. The first detection of anti-N antibodies was carried out in all samples by using ID Screen^®^ Schmallenberg virus Competition Multi-species (ID.vet) following the manufacturer’s protocol. For the Virus Neutralization Test (VNT), the serum samples were heat inactivated and were 12 times two-fold diluted in Eagle’s Minimum Essential Medium (EMEM), starting at ½ in 96-well plates. Approximately 100–200 TCID_50_ SATV or SHAV was then added to each diluted serum before overnight incubation at 37°C. After the overnight incubation, the serum/virus mixture was added to 96-well plates where 4 × 10^4^ BHK-21 were cultured 24 h before inoculation. The results of the VNT were expressed as the effective dilution neutralizing 50% of the challenge virus (ED_50_) and were considered positive if log2 ED_50_ was >2.

As previously shown by Causey et al. [18], we demonstrated an absence of cross-neutralization between animals infected with SHAV and SATV. In addition, when serum samples of sheep were infected (naturally or experimentally) with SBV, these samples neutralized SATV exclusively and did not neutralize SHAV.

### Viral titre and viral growth kinetics

The viral titre of the two parental viruses and all the reassortants was determined by calculating the TCID_50_/mL (Tissue Culture Infectious Dose 50). 24 h before inoculation, 4 × 10^4^ BHK-21 cells were cultured in 96-well plates. The viruses were diluted (10^1^–10^10^) in EMEM containing 1% of FBS. The growth medium was removed, and each well was inoculated with a 50 µL volume of each dilution of the viruses. After incubation at 37°C for two hours, 100 µL of complete GMEM was added to each well and the cells were transferred at 37°C. Seventy-two hours later, the growth medium was removed and the cells were fixed and coloured with crystal violet. The 50% infective dose was calculated by the Reed and Muench method.

In order to compare the replication capacities of the parental viruses in both cellular contexts or to calculate the reassortant viral growth kinetics, triplicate infections of BHK-21 and/or KC cells were performed in 6-well plates with a similar infection dose (MOI 0.01). At 0, 2, 12, 24, 36, 48 and 72 h post infection, 200 µL of supernatant was collected and the titre was determined for each sample as described above.

### Co-infection experiments and competition assay

Monolayers of BHK-21 cells were prepared in 25 cm^2^ flasks (1 × 10^6^ cells) one day before the co-infection assays. SHAV and SATV viruses were mixed in equal proportions and were diluted in EMEM with 1% FBS to obtain appropriate titre for infection at MOI 0.01 or 10/well corresponding to MOI 0.005 or MOI 5 of each virus. Cells were inoculated using 800 µL of the medium dilutions containing the viruses and were incubated at 37°C. Two hours later, the infecting supernatant was removed, cells were washed three times and 5 mL of complete medium was added to the overlay. 48 h after infection, the supernatant was collected and frozen at −80°C.

The co-infection supernatant was used to perform the competition assay in BHK-21 cells. Dilutions 10^−4^ of the supernatant were used to infect new BHK-21 monolayers. The infections were performed over 48 h and 10 successive passages in BHK-21 cells were carried out with this dilution rate. At each passage, the infectious supernatants were collected and frozen at −80°C before being reused for a new infection.

In order to infect the KC cells, a different strategy was used than for the BHK-21 cells. To promote the infection of these cells, KC cells were prepared in 25 cm^2^ flasks (4 × 10^6^ cells) and were infected simultaneously. SHAV and SATV viruses were mixed in equal proportions and were diluted in Schneider medium with 1% FBS to obtain the appropriate titre for infection at MOI 0.01 or 10/well corresponding to MOI 0.005 or MOI 5 of each virus respectively.

After counting, the KC cells were centrifuged and the supernatant was removed. Cell pellets were re-suspended in the appropriate volume of Schneider medium with 1% FBS, and 500 µL was distributed in 25 cm^2^ flasks. The viral inoculum (1 mL) was immediately mixed with the cells, and the flasks were incubated at 25°C. Three hours later, the infecting supernatant was removed, cells were washed three times and 5 mL of complete Schneider medium was added to the overlay. 72 h after infection, the supernatant was collected and frozen at −80°C.

Owing to the lower infection capacities and the non-lytic phenotypes of the Simbuviruses in these cells, the supernatant was not used to propagate viruses for the competition assay in KC cells. Instead of using an infectious supernatant, the infected cells were successively amplified to allow the propagation of viruses. Between each passage, the supernatant was collected and frozen at −80°C and infected cells were split in a ¼ ratio and cultured in a new 25cm^2^ flask with fresh complete Schneider medium.

For competition assay using both BHK-21 and KC cells, the protocol was similar to the one described for BHK-21. In this case, the amplification of the viral population produced in BHK-21 cells was appropriate to propagate the infection in KC cells.

### Isolation and screening of progeny viruses

To isolate and amplify the progeny viruses after the co-infection experiment, a limiting dilution assay was performed.

BHK-21 cells were prepared in 96-well plates one day in advance (4 × 10^4^ cells per well) and were infected with 50 µL per well of appropriate dilution of inoculum in an EMEM medium containing 1% FBS. One to three 96-well plates were infected with one dilution. Isolated viruses were collected if the proportion of infected wells was less than 25% on a total 96-well plate, and were amplified by inoculation into BHK-21 cells grown in 24-well plates. After three days, the supernatants were collected and frozen at −80°C before further analyses.

### Characterization of progeny viruses

After the amplification of each isolated sample, the total RNA from the supernatant was extracted with the QiAmp Viral Mini Kit (Qiagen). To determine the origin of each segment, the viral RNA was reverse transcribed with random primer by using SuperScript III reverse transcriptase (Invitrogen). The resulting cDNAs were amplified by discriminative PCR with different sets of primers (Table S1). One discriminative set of primers was made up of one universal primer (located on the segments’ extremities) and by one SHAV specific primer or one SATV specific primer located near to each extremity of each segment (Figure 3(a)). Two PCRs (one SHAV specific and one SATV specific) using GoTaq polymerase (Promega) were performed on each segment extremity. The PCR-amplification protocol consisted of 3 min at 94°C, followed by 32 cycles of 30 s at 94°C, 30 s at 55°C and 45 s at 72°C, and a terminal incubation at 72°C for 7 min. The genotype of each virus was determined by an analysis of the PCR migration (Figure 3(b)).

### Plaque assays

Plaque assays were performed as previously described [45], with three different sets of isolates of each reassortant. To determine the relevance of the differences in plaque-size, 50 randomly-selected plaques were analysed in each assay with ImageJ software (http://rsb.info.nih.gov/ij/). A statistical method adapted for plaque sizes analysis was used in order to take into account the population variations and the distribution [46]. To this end, the data were analysed using Kolmogorov Smirnov statistics as previously described [47].

An arbitrary score was given to the two parental viruses. SHAV, which has a phenotype large plaque, had a score of 4, and SATV, which has a phenotype small plaque, had a score of 2. Thus, there were five possibilities. If the reassortant harboured the same plaque size as one of the two parental viruses, this reassortant obtained a score equal to the parental score, 2 or 4. If the reassortant harboured plaque sizes smaller than the SHAV and SATV parental viruses, the score was equal to 1. If the reassortant harboured plaque sizes smaller than SHAV and bigger than SATV, the score was 3. If the reassortant harboured plaque sizes bigger than SHAV and SATV, the score was 5.

### Statistical analysis

The growth kinetics of each reassortant were compared each other’s using ANOVA2 statistics.

The plaque sizes of each reassortant were compared with the parental ones (SHAV and SATV) using Kolmogorov–Smirnov statistics.

An ANOVA test was performed to compare the scores of plaque sizes of the six reassortants together and a Tukey test was performed to compare each reassortant against every other reassortant. The Welch’s *t*-test was performed to compare groups of reassortants according to their segment origin.

## Supplementary Material

Supplemental Material
