# Supplementary material for: Host-dependence of in vitro reassortment dynamics among the Sathuperi and Shamonda Simbuviruses
Source: Emerg Microbes Infect. 2019 Mar 21;8(1):381–95. doi: 10.1080/22221751.2019.1586410 (PMC6455117; doi:10.1080/22221751.2019.1586410)
Supplement: Supplemental Material [file TEMI_A_1586410_SM2501.zip › Supplementary Material/TableS1.docx]

|  | **S segment 3' gRNA** | | | **S segment 5'gRNA** | | |
| --- | --- | --- | --- | --- | --- | --- |
|  | **universal primer** | **SATV primer** | **SHAV primer** | **universal primer** | **SATV primer** | **SHAV primer** |
| **Primer name** | For-S-univ | Rev-S-SATV | Rev-S-SHAV | Rev-S-univ | For-S-SATV | For-S-SHAV |
| **SATV binding** | AGTAGTGAGCTCCACTATTAAC \|\|\|\|\|\|\|\|o\|\|\|\|\|\|\|\|\|\|\|\|\| | CACTTGCTTCAGCCAACTTA \|\|\|\|\|\|\|\|\|\|\|\|\|\|\|\|\|\|\|\| | TCTCGATAACCAGTGGGTAG  \|o\|\|\|\|\|\|\|\|o\|\|o\|\|o\|\|o | AGTAGTGTTCTCCACTTATTAACTATC \|\|\|\|\|\|\|\|\|\|\|\|\|\|\|\|\|\|\|\|\|\|\|\|\|\|\| | TTAAGGCTGCGTTCAACTCC \|\|\|\|\|\|\|\|\|\|\|\|\|\|\|\|\|\|\|\| | TTGATGCTTTTGACTTCTACCCA \|\|\|\|\|\|\|\|\|\|\|\|\|o\|\|o\|\|o\|\|o |
| **Primer sequence (5'-3')** | AGTAGTGAACTCCACTATTAAC | CACTTGCTTCAGCCAACTTA | TTTCGATAACTAGCGGATAA | AGTAGTGTTCTCCACTTATTAACTATC | TTAAGGCTGCGTTCAACTCC | TTGATGCTTTTGATTTTTATCCG |
| **SHAV binding** | \|\|\|\|\|\|\|\|\|\|\|\|\|\|\|\|\|\|\|\|\|\| AGTAGTGAACTCCACTATTAAC | \|\|\|\|o\|\|\|\|\|\|\|\|o\|oo\|\|o CACTAGCTTCAGCTAGTTTG | \|\|\|\|\|\|\|\|\|\|\|\|\|\|\|\|\|\|\|\| TTTCGATAACTAGCGGATAA | \|\|\|\|\|\|\|\|\|\|\|\|\|\|\|\|\|\|\|\|\|\|\|\|\|\|\| AGTAGTGTTCTCCACTTATTAACTATC | \|o\|\|\|\|\|\|\|\|o\|\|\|\|\|o\|\|o TAAAGGCTGCTTTCAATTCT | \|\|\|\|\|\|\|\|\|\|\|\|\|\|\|\|\|\|\|\|\|\|\| TTGATGCTTTTGATTTTTATCCG |
| **position (amplicon length)** | 1→22 | 389→370 (389 bp) | 521→502 (521 bp) | 840→814 (SATV)/ 839→813 (SHAV) | 633→652 (207 bp) | 486→508 (353) |
|  | **M segment 3'gRNA** | | | **M segment 5'gRNA** | | |
|  | **universal primer** | **SATV primer** | **SHAV primer** | **universal primer** | **SATV primer** | **SHAV primer** |
| **Primer name** | For-M-univ | Rev-M-SATV | Rev-M-SHAV | Rev-M-univ | For-M-SATV | For-M-SHAV |
| **SATV binding** | AGTAGTGAACTACCACAAT \|\|\|\|\|\|\|\|\|\|\|\|\|\|\|\|\|\|\|\|\|\| | TATTTAGAACAAGGAAAGTACCC ooo\|oooo\|oo\|oo\|oooooooo | CCAGTAATAGAATAATGGTTGAG \|\|\|\|\|\|\|\|\|\|\|\|\|\|\|\|\|\|\|\|\|\|\| | AGTAGTGTTCTACCA \|\|\|\|\|\|\|\|\|\|\|\|\|\|\|\|\|\|\|\|\|\| | TCTCTCTTCATCCTGATCTTGACTC \|\|\|\|\|\|\|\|\|\|\|\|\|\|\|\|\|\|\|\|\|\|\|\|\| | TTAGTGTAATATTCCAATCTCTAT oo\|ooo\|\|oooooooo\|\|oooo\|o |
| **Primer sequence (5'-3')** | AGTAGTAGTGAACTACCAMAAT | CGATATCTAATATCATGAAGGAG | CCAGTAATAGAATAATGGTTGAG | GAGTAGTAGTAGTGTTCTACCA | TCTCTCTTCATCCTGATCTTGACTC | GAATCATAGGGCCAATATGGAAAG |
| **SHAV binding** | \|\|\|\|\|\|\|\|\|\|\|\|\|\|\|\|\|\|\| agtAGTAGTGAACTACCAAAAT | \|\|\|\|\|\|\|\|\|\|\|\|\|\|\|\|\|\|\|\|\|\|\| CGATATCTAATATCATGAAGGAG | oo\|oo\|oooooooo\|\|oo\|oooo GTACCAGATATTCGATAATGATT | \|\|\|\|\|\|\|\|\|\|\|\|\|\|\|  AGTAGTGTTCTACCA | \|oooooo\|o\|\|oo\|o\|\|oo\|\|\|ooo TTCTCTGTTATTTTAATAGTGAAAG | \|\|\|\|\|\|\|\|\|\|\|\|\|\|\|\|\|\|\|\|\|\|\|\| GAATCATAGGGCCAATATGGAAAG |
| **position (amplicon length)** | 1→19 | 333→311 (333 bp) | 460→438 (460 bp) | 4365→4351 (SATV) / 4315→4301 (SHAV) | 4122→4146 (243 bp) | 4051→4074 (264 bp) |
|  | **L segment 3'gRNA** | | | **L segment 5'gRNA** | | |
|  | **universal primer** | **SATV primer** | **SHAV primer** | **universal primer** | **SATV primer** | **SHAV primer** |
| **Primer name** | For-L-univ | Rev-L-SATV | Rev-L-SHAV | Rev-L-univ | For-L-SATV | For-L-SHAV |
| **SATV binding** | AGTAGTGTACCCCTAATTAC aaa\|\|\|\|\|\|\|\|\|\|\|\|\|\|\|\|\|\|\|\| | CCATATGTATGGTCTGTTGAG \|\|\|\|\|\|\|\|\|\|\|\|\|\|\|\|\|\|\|\|\| | CTTGACATGGATTGTATCTCTT  \|\|\|\|\|\|\|\|\|o\|\|o\|\|\|\|\|\|\|oo | AGTAGTGTGCCCCTAATTACATG \|\|\|\|\|\|\|\|\|\|\|\|\|\|\|\|\|\|\|\|\|\|\| | ACATCTACCACAATTTCCAAATC \|\|\|\|\|\|\|\|\|\|\|\|\|\|\|\|\|\|\|\|\|\|\| | TAACATGGATTTTGAATATGCTG \|o\|\|o\|\|\|\|\|o\|\|o\|\|o\|o\|\|\|o |
| **Primer sequence (5'-3')** | AGTAGTAGTGTACCCCTAATTAC | CCATATGTATGGTCTGTTGAG | CTTGACATGAATAGTATCTCGC | AGTAGTGTGCCCCTAATTACATG | ACATCTACCACAATTTCCAAATC | TGACTTGGATCTTAAACAAGCTA |
| **SHAV binding** | \|\|\|\|\|\|\|\|\|\|\|\|\|\|\|\|\|\|\|\| aaaAGTAGTGTACCCCTAATTAC | \|\|\|\|\|\|\|o\|\|\|o\|\|o\|\|\|\|\|o CCATATGCATGATCAGTTGAT | \|\|\|\|\|\|\|\|\|\|\|\|\|\|\|\|\|\|\|\|\|\| CTTGACATGAATAGTATCTCGC | \|\|\|\|\|\|\|\|\|\|\|\|\|\|\|\|\|\|\|\|\|\|\| AGTAGTGTGCCCCTAATTACATG | \|o\|\|\|\|\|o\|\|o\|\|\|\|\|o\|\|\|o\|o ATATCTATCATAATTTTCAAGTT | \|\|\|\|\|\|\|\|\|\|\|\|\|\|\|\|\|\|\|\|\|\|\| TGACTTGGATCTTAAACAAGCTA |
| **position (amplicon length)** | 1→20 | 326→306 (326 bp) | 444→423 (444 bp) | 6882→6860 | 6518→6540 (364 bp) | 6425→6447 (457 bp) |
