# Supplementary figures and images for: Host-dependence of in vitro reassortment dynamics among the Sathuperi and Shamonda Simbuviruses
Source: Emerg Microbes Infect. 2019 Mar 21;8(1):381–95. doi: 10.1080/22221751.2019.1586410 (PMC6455117; doi:10.1080/22221751.2019.1586410)

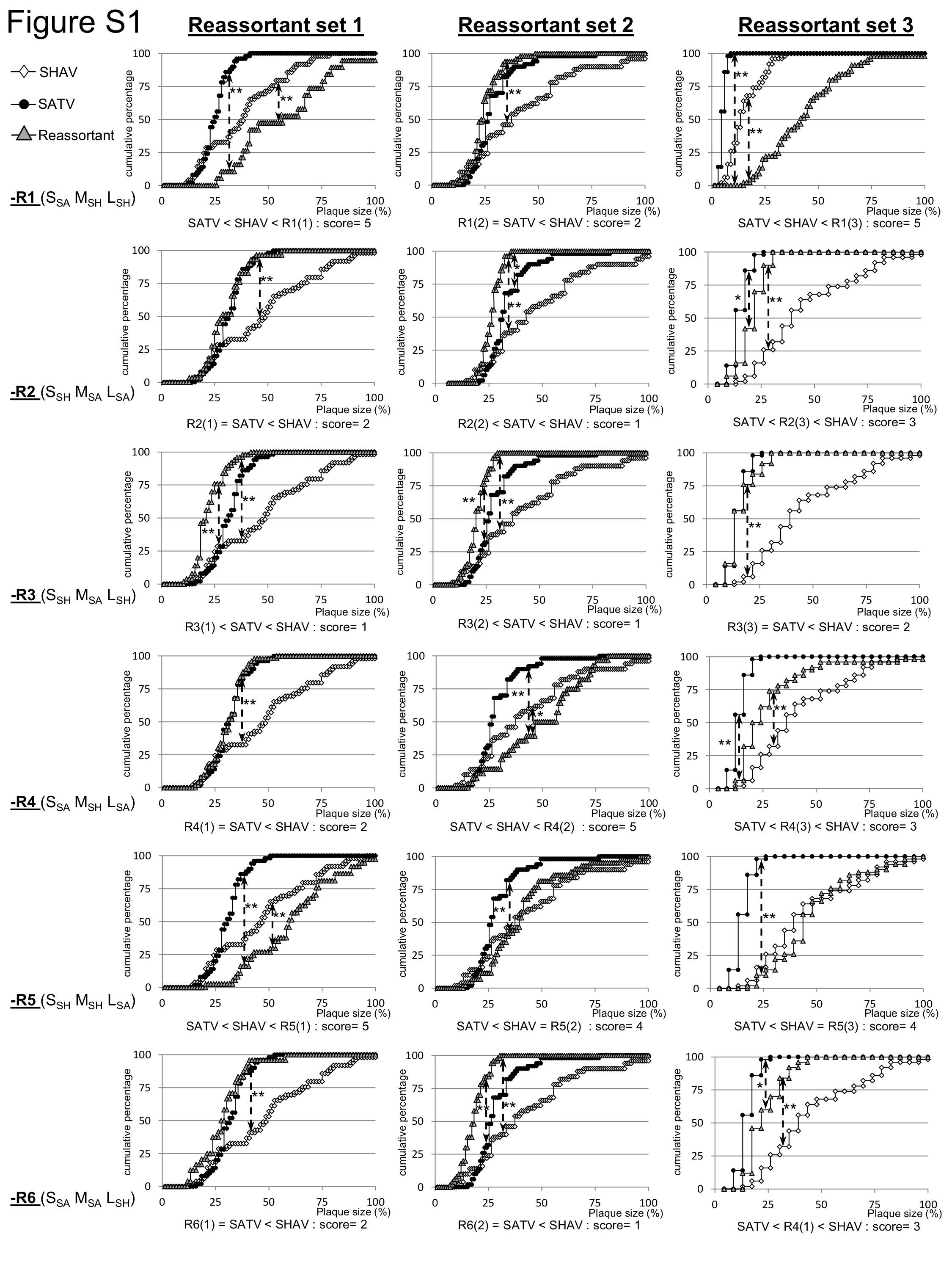

Supplement: Supplemental Material [file TEMI_A_1586410_SM2501.zip › Supplementary Material/figure S1 TIFF.tiff]

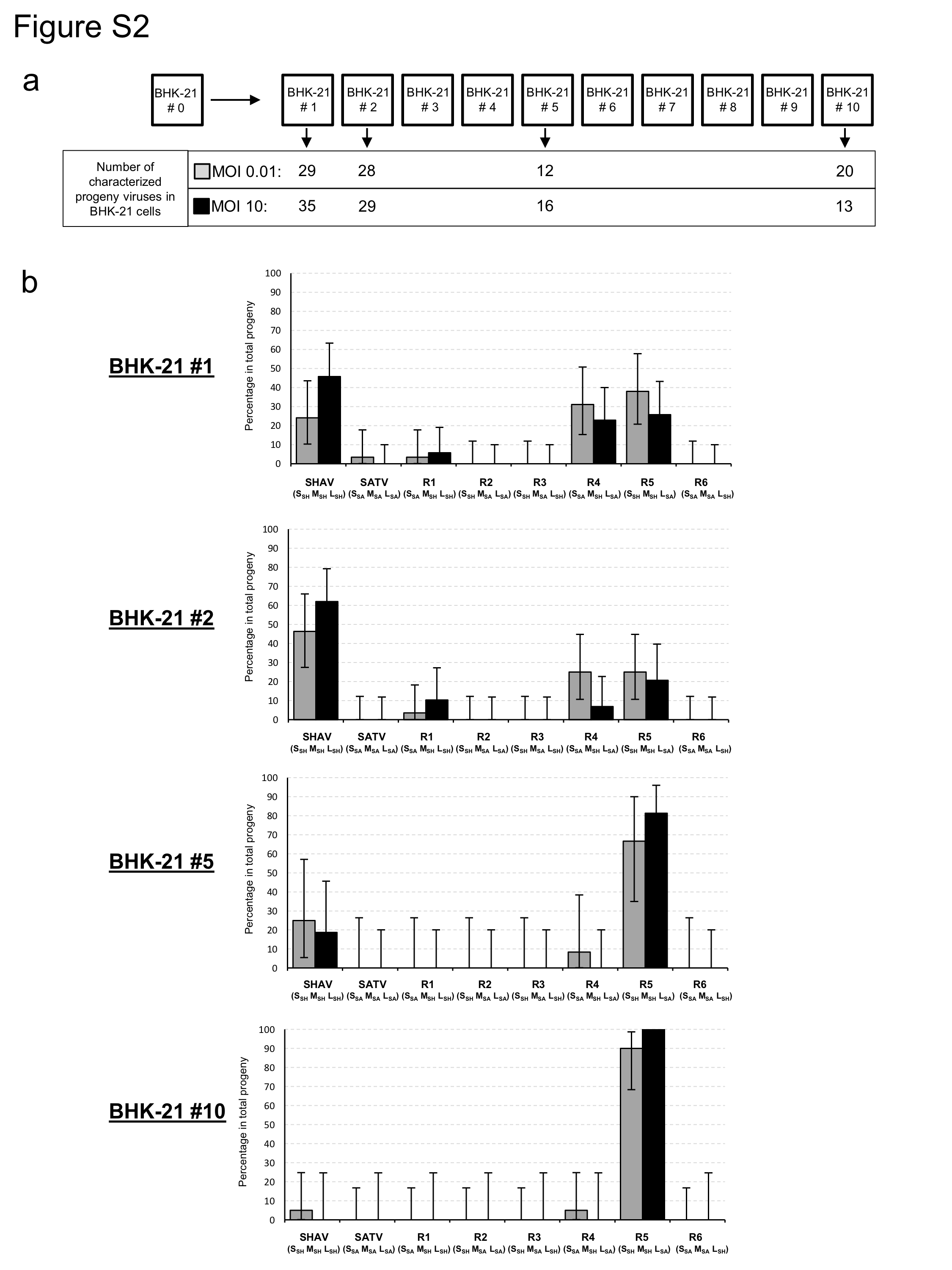

Supplement: Supplemental Material [file TEMI_A_1586410_SM2501.zip › Supplementary Material/figure S2 TIFF.tiff]
